# The epidemiology of blindness in children: changing priorities

**Published:** 2018-02-08

**Authors:** Clare Gilbert, Richard Bowman, Aeesha NJ Malik

**Affiliations:** 1 Co-director: nternational Centre for Eye Health, London School of Hygiene & Tropical Medicine, London, UK.; 2 Honorary Clinical Consultant: nternational Centre for Eye Health, London School of Hygiene & Tropical Medicine, London, UK.; 3 Clinical Research Fellow: International Centre for Eye Health, London School of Hygiene & Tropical Medicine, London, UK.

**The number of children who are blind from eye conditions (excluding refractive error) is falling in all regions. To continue this encouraging trend, comprehensive eye care needs to be strengthened by improving referral mechanisms and counselling parents at every step.**

**Figure:**
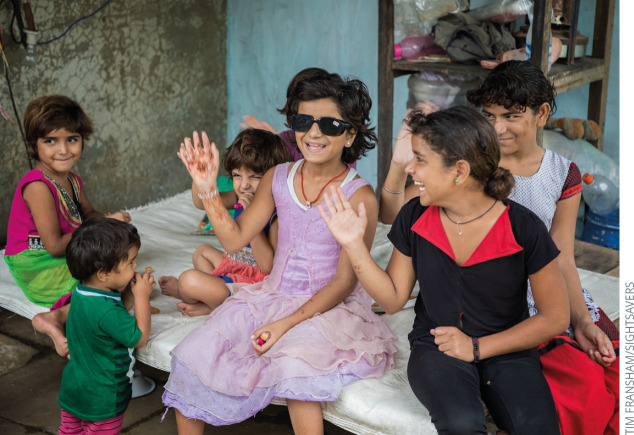
A young girl after cataract surgery. Cataract is now the main cause of avoidable blindness in many low-income countries. INDIA

Nearly a third of the very first issue of the *Community Eye Health Journal* was about blinding eye diseases in children. One article described an Indian study about improving mothers' knowledge so that they could prevent eye conditions in their children. The other article also focused on the major causes of corneal scarring in children: vitamin A deficiency (VAD) and measles infection.

The issue was published two years before the first workshop on childhood blindness, which was initiated by the World Health Organization (WHO) and held in London in 1990. The workshop report reviewed what was then known about the prevalence and causes of blindness in children and estimated that there were 1.5 million blind children worldwide.^1^ At that time, corneal scarring – principally from VAD – was estimated to be responsible for 50–70% of blindness among children in low-income settings. Every year, there were 350,000 new cases of xerophthalmia; an estimated 60% of these children would die within a year of becoming blind. Measles infection was recognised as an important cause of vitamin A deficiency.

## Measles and vitamin A deficiency

**Figure 1:**
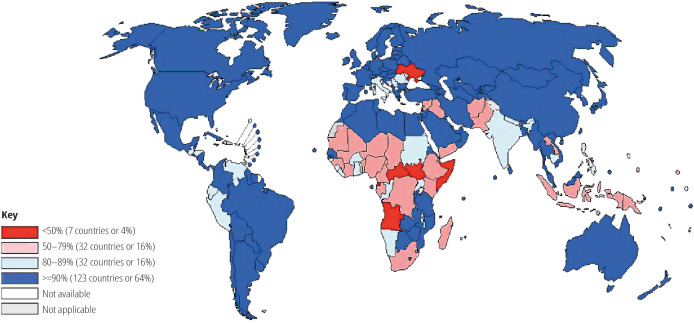
Measles immunisation coverage: first dose, 2015^2^

Much has happened since then. Measles immunisation coverage (the proportion of children who have been immunised, expressed as a percentage) has increased in many countries to over 80% (Figure 1), with a marked reduction in the number of measles cases. In 2015, however, there were still estimated to be over 134,000 deaths from measles.^2^ As can be seen in Figure 1, most countries in sub-Saharan still have immunisation coverage below the target of 80%.

**Figure:**
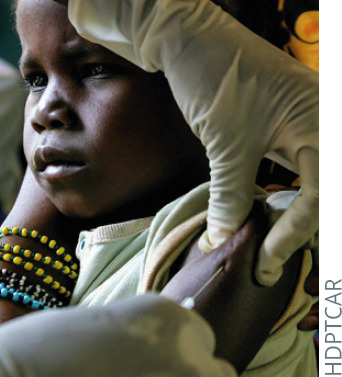
Measles vaccination. CENTRAL AFRICAN REPUBLIC

**Figure 2:**
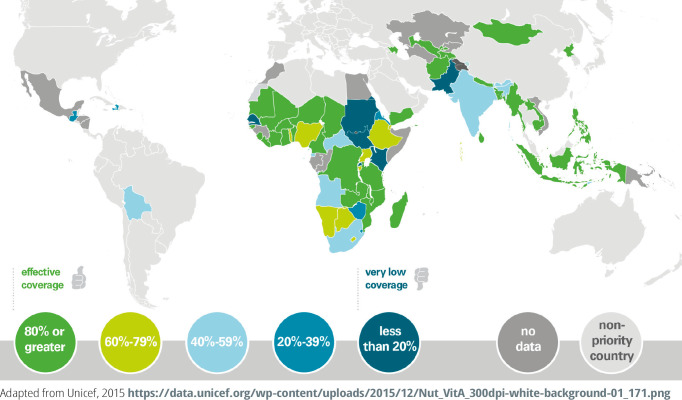
Vitamin A supplementation coverage, 2014

Adapted from Unicef, 2015 **https://data.unicef.org/wp-content/uploads/2015/12/Nut_VitA_300dpi-white-background-01_171.png**

**Figure:**
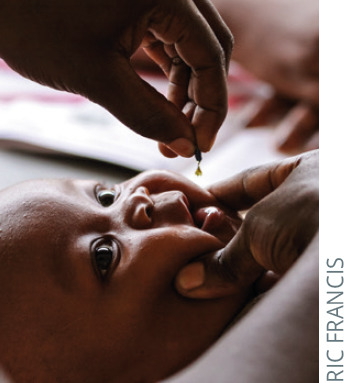
A four-month-old baby receives a dose of vitamin A. KENYA

In the late 1980s it became clear that children with xerophthalmia had a higher mortality rate than children without the condition. This important finding led to many clinical trials of vitamin A supplementation. Trials have shown that, in communities with less than adequate nutrition, supplementation of children aged 6–59 months reduces child mortality and morbidity, and also reduces the ocular signs of vitamin A deficiency.^3^ Today, vitamin A supplementation (two doses per year for children aged 6–59 months) is being implemented in child health programmes in low-income countries. However, as with measles, vitamin A supplementation coverage is below 80% in many countries (Figure 2).^4^ It is important to note that other approaches to improve the nutritional status of children, including their vitamin A intake, should go hand-in-hand with supplementation. These approaches include supplemental feeding, fortification of commonly consumed foods such as oil and sugar, and breeding crops so they have a higher vitamin A content (known as biofortification).

What impact have these large-scale public health initiatives had on the causes of blindness in children in low income countries? The simple answer is that they have had a major impact, with a marked decline in the proportion of blindness due to corneal scarring in many countries. However, we must not become complacent. In some countries, such as Ethiopia, corneal scarring remains the commonest cause of blindness^5^, and sub-clinical vitamin A deficiency in children remains endemic in many countries. Indeed, UNICEF estimates that 33% of preschool-age children and 15% of pregnant women in low income countries do not have enough vitamin A in their daily diet, and that 5.2 million preschool-age children have clinical vitamin A deficiency. More needs to be done to increase awareness in communities about the need for a vitamin A-rich diet, and to improve the coverage of vitamin A supplementation.

## Changes in demography and under-five mortality rates

**Figure 3:**
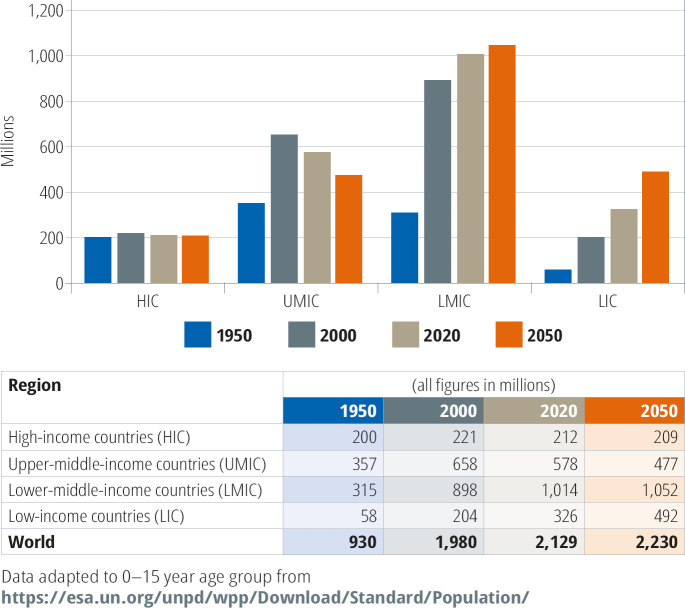
Child population (millions) aged 0–15 years, from 1950 to 2050, by region

What else has changed since 1988? The number of children in the world aged 0–15 years has increased from around 930 million in 1950 to 2 billion today. But the rate of increase is slowing, largely as a result of socio-economic development. The number of children has declined in upper-middle-income countries (UMIC) but is projected to continue to increase in low-income countries (LIC).^6^ See Figure 3.

**Figure 4:**
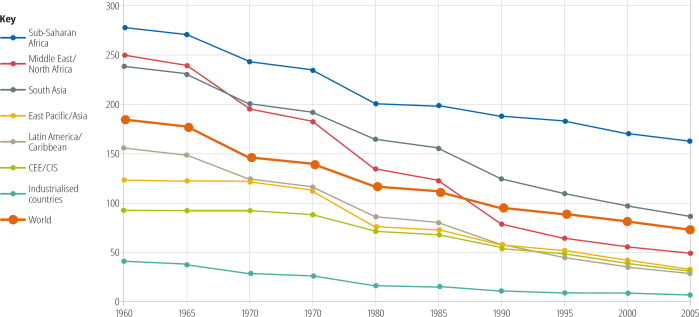
Under-five mortality rates globally and by region*

In the mid-1990s, it became clear that the prevalence of blindness in children is associated with under-five mortality rates: it is higher in countries with high under-five mortality rates, and low in countries with low under-five mortality rates. As can be seen in Figure 4, under-five mortality rates are declining in all regions. In 2005, sub-Saharan Africa had the highest under-five mortality rate compared with other regions.

Data adapted to 0–15 year age group from **https://esa.un.org/unpd/wpp/Download/Standard/Population/**

The association between the prevalence of blindness in children and under-five mortality rates has been used to update the estimate of the number of blind children in the world.^7^ The estimates are as follows:

- 1.5 million in 1990
- 1.4 million in 1999
- 1.26 million in 2010
- 1.14 million in 2015.

**Figure 5:**
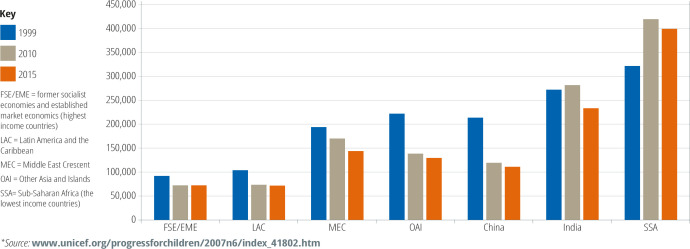
Regional variation in the estimates of blind children between 1990 and 2015*

*Source: **www.unicef.org/progressforchildren/2007n6/index_41802.htm**

There has been a reduction of 24% in the number of blind children since 1990, despite the overall increase in the child population. Why is this happening? Better control of measles and vitamin A deficiency, which are both important causes of blindness, are contributing to declining under-five mortality rates (Figure 4).^8^ However, the overall decline in the number of blind children globally hides regional differences in the change (Figure 5). In sub-Saharan Africa, the estimated number increased between 1999 and 2010, but is now declining.

## Childhood cataract

In many low-income countries where corneal scarring has declined, cataract has become the commonest cause of avoidable blindness in children. Much has been done to establish tertiary eye care centres with a well trained and equipped team, and many of the larger countries now have several such child eye care centres. One of the main challenges is that affected children often present very late for surgery due to lack of awareness and cultural, social and economic barriers, which compromises the visual outcomes. There is also some evidence that, in Asian countries, girls with bilateral cataract do not access services at the same rate as boys.^9^ Another study from Bangladesh showed that children with better visual outcomes after cataract surgery were more likely to be in school, so cataract surgery contributes towards the Sustainable Development Goals regarding gender and education.^10^ More needs to be done to improve access, to ensure that children attend for follow-up after surgery, and to provide low vision services for the children who do not have good visual outcomes.

**Figure 6:**
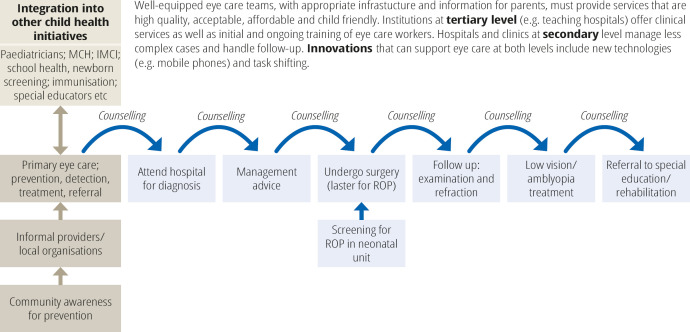
Comprehensive clinical care for children at secondary and tertiary level (adapted from Aravind Eye Care System)

## Prematurity and retinopathy

Another major change over the last 20 years is that services for preterm infants have expanded dramatically, as governments realise that Under-five mortality rates will remain high unless neonatal and infant mortality rates are brought down. Neonatal care initially expanded in upper-middle income countries, particularly in Latin America and the Caribbean, in the former socialist economies, and subsequently in many Asian countries. Neonatal care has just begun to expand in Africa. However, in most countries, policies and resources were not put in place to control retinopathy of prematurity, which has led to the ‘third epidemic’ of blindness due to retinopathy of prematurity.^11^ A recent estimate of the annual number of infants becoming blind or visually impaired from retinopathy of prematurity shows that every region is affected, with 32,300 new cases every year.^12^ In middle-income countries, retinopathy of prematurity is often the commonest cause of avoidable blindness. Many of these countries have or are responding by establishing screening and treatment programmes. However, more needs to be done to increase the coverage and quality of screening and treatment and to improve the quality of neonatal care, as this will reduce the incidence of retinopathy of prematurity needing treatment. Advocacy is needed to raise awareness among ministries of health and other agencies engaged in child health. The goal is to ensure that policies and programmes, with guidelines and resources, are put in place to reduce this potentially avoidable cause of blindness.

## Cerebral visual impairment

Cerebral visual impairment (due to damage to the visual pathways in the brain) is the leading cause of severe visual impairment and blindness in children in high-income countries. It is also an emerging cause in low-income countries, where a relatively high proportion is attributable to perinatal factors and so potentially avoidable through better perinatal care.^13^ Cerebral visual impairment may be missed because it usually affects children who also have other disabilities such as cerebral palsy or learning difficulties. A community-based study of cerebral palsy in Bangladesh showed that a third of children had reduced visual acuity and over half had visual perception problems which adversely affected their quality of life.^14^

## Going forward

To improve child eye health and reduce disability, comprehensive services are needed at community, primary, secondary, and tertiary level, working alongside low vision, special education and rehabilitation services. Good referral mechanisms are needed to provide a continuum of care between all services (Figure 6).

Raising awareness in the community about eye diseases, and how they can be prevented, is very important, as was described in the first edition of the *Community Eye Health Journal.* At the primary level, staff providing services for mothers and young children need to know what they can do to prevent, detect and treat and eye diseases (pp. 78–79).^15^ Eye care at secondary level needs to be strengthened to manage less complex cases and to follow children up after surgery. Screening and treatment of ROP can be undertaken by ophthalmologists at secondary or tertiary levels. Counselling parents at every step is of vital importance as it will help them to understand what to do and the important role they play.

Much is being done to improve tertiary eye care for children, but more tertiary centres are needed; ideally one per ten million population.^16^ Greater emphasis is also needed on the other levels of service delivery, special education and rehabilitation; and the referral networks between them.

## References

[B1] Imdad A, et al. Vitamin A supplementation for preventing morbidity and mortality in children from six months to five years of age. Cochrane Database Syst Rev. 2017;11(3):CD008524. 10.1002/14651858.CD008524.pub3 PMC6464706 28282701

[B2] Mulusew A, et al. Causes of severe visual impairment and blindness in students in schools for the blind in Northwest Ethiopia. BMJ Glob Health 2017;2:e000264. 10.1136/bmjgh-2016-000264 PMC5717965 29225928

[B3] Negretti GS, et al. Cataract surgery outcomes in Bangladeshi children. Ophthalmol 2015;122(5):882–7. 10.1016/j.ophtha.2015.01.013 25704321

[B4] Aghaji A, et al. Causes and emerging trends of childhood blindness: findings from schools for the blind in Southeast Nigeria. Br J Ophthalmol 2015;99:727–31. 25472948 10.1136/bjophthalmol-2014-305490

[B5] Malik ANJ Mafwiri M Gilbert C. Integrating primary eye care into global child health policies. Arch Dis Child. 2017 Oct 7. Epub ahead of print. 10.1136/archdischild-2017-313536 PMC5865509 28988214

